# Instantaneous frequency from Hilbert-Huang transformation of digital volume pulse as indicator of diabetes and arterial stiffness in upper-middle-aged subjects

**DOI:** 10.1038/s41598-018-34091-6

**Published:** 2018-10-25

**Authors:** Hai-Cheng Wei, Ming-Xia Xiao, Hong-Yu Chen, Yun-Qin Li, Hsien-Tsai Wu, Cheuk-Kwan Sun

**Affiliations:** 1 School of Electrical and Information Engineering, North Minzu University, No. 204 North – Wenchang St., Xixia District, Yinchuan, Ningxia 750021 China; 2 School of Computer and Information, Hefei University of Technology, No. 193, Tunxi Rd., Hefei, Anhui 230009 China; 3 Department of Electrical Engineering, National Dong Hwa University, No. 1, Sec. 2, Da Hsueh Rd., Shoufeng, Hualien 97401 Taiwan; 4 Department of Emergency Medicine, E-Da Hospital, I-Shou University School of Medicine for International Students, No. 1, Yida Road, Jiaosu Village, Yanchao District, Kaohsiung City, 82445 Taiwan

## Abstract

To investigate the value of decomposed short-time digital volume pulse (DVP) signals in discerning systemic vascular anomaly in diabetic patients, demographic and anthropometric parameters, serum lipid profile, fasting blood glucose and glycated hemoglobin (HbA1c) levels were obtained from 29 healthy adults (Group 1) and 29 age-matched type 2 diabetes mellitus patients (Group 2). Six-second DVP signals from right index finger acquired through photoplethysmography were decomposed using ensemble empirical mode decomposition. Using one intrinsic mode function (IMF5), stiffness index (SI) and instantaneous energy of maximal energy (f_Emax_) were obtained. Other indicators of arterial stiffness, including electrocardiogram-pulse wave velocity of foot (ECG-PWV_foot_), crest time (CT) and crest time ratio (CTR), were obtained from the testing subjects for comparison. The mean body weight, body mass index, waist circumference, HbA1c and fasting blood sugar levels were higher in Group 2 than those in Group 1, whereas values of systolic and diastolic blood pressure were lower in Group 2 than those in Group 1. SI and f_Emax_ were significantly higher in Group 2 than those in Group 1. Moreover, f_Emax_ was positively associated with HbA1c concentration, CT and SI in Group 2 (*p* < 0.05) but not in Group 1. When all subjects were considered, f_Emax_ was highly significantly associated with HbA1c and fasting blood sugar levels, and SI (all *p* < 0.001). After Hilbert-Huang transformation, short-time DVP signals could give significant information on arterial stiffness and vascular anomaly in diabetic patients.

## Introduction

Not only is cardiovascular disease a major killer in the developed world, but it is also a non-communicable disease that poses an ever-increasing health threat in the developing countries^1^. Besides, the gruesome disabilities for the survivors result in staggering social and economic burden worldwide^2^. Accordingly, prevention and early detection of the disease in high-risk patients such as those with diabetes have become important issues in preventive medicine and public health^3^.

Cost-effective and non-invasive approaches to early detection of cardiovascular disease have been widely investigated. Parameters including pulse wave velocity (PWV)^4^, cardio-ankle vascular index (CAVI)^5^, and ankle-brachial blood pressure index (ABI)^6^ have been validated as non-invasive assessment tools for vascular health. Despite their non-invasiveness, unfavorable factors including the cost of the equipment, the need for technical assistance during measurement, the relatively long time for data acquisition as well as their hospital-based settings hinder their popularity as screening tools. With the advance of artificial intelligence and computational technologies, it is now possible to analyze physiological signals using portable electronic equipment with built-in computational programs to produce affordable devices for daily use^7^.

Arterial waveform analysis previously focused on direct observation of changes in waveform signals with the time domain as in the computation of stiffness index (SI) and crest time (CT)^8^. Digital volume pulse (DVP) acquired through photoplethysmography (PPG) has gained much popularity in assessing vascular health because of its non-invasiveness, convenience, and low cost^9^. After detrending and eliminating background noises using ensemble empirical mode decomposition (EEMD) (i.e., a component of HHT), DVP signals from the finger have been previously shown to successfully differentiate between normal and diabetic subjects using the multiscale cross-approximate entropy approach^10^.

Since conventional Fourier analysis only displays data as sine and cosine functions, it cannot help in the computation of stiffness index (SI) that requires discernible systolic and diastolic digital volume pulse (DVP) waveforms which are often difficult to obtain in subjects with systemic diseases such as diabetes^11^. Ensemble empirical mode decomposition (EEMD), which is the first component of Hilbert-Huang transformation (HHT), separates acquired physiological signals into a set of distinct physiological information known as intrinsic mode functions (IMFs)^12^. It has been shown that IMF5 best represents DVP waveforms through which SI can be calculated^13^. It is our hypothesis that physiological information hidden in the inseparable signal of an IMF as reflected in the energy-frequency width spectrum can be revealed through Hilbert–Huang spectrum analysis, which is the second component of HHT. Therefore, the present study aimed at assessing the significance of instantaneous frequency of maximal energy (f_Emax_) derived from marginal Hilbert spectrum and instantaneous frequency in vascular health through comparing the index in diabetes subjects with that in healthy volunteers. Established indices of arterial stiffness including SI, electrocardiogram-pulse wave velocity of foot (ECG-PWV_foot_)^14^, crest time (CT) and crest time ratio (CTR)^11^ were also acquired from the testing subjects for comparison.

## Results

### Baseline characteristics of study subjects

Diabetic group (Group 2) had significantly higher mean body weight, body mass index (BMI), and waist circumference (all *p* < 0.05) as well as concentrations of glycated hemoglobin (HbA1c) and fasting blood sugar (both *p* < 0.001) than those in the non-diabetic group (Group 1) (Table 1). On the other hand, the systolic (SBP) and diastolic blood pressure (DBP) were lower in Group 2 than those in Group 1 (both *p* < 0.05).

**Table 1:** Comparisons of demographic, anthropometric, serum biochemical, and computational parameters between diabetic and non-diabetic subjects.

	Group 1Male/Female (14/15)	Group 2Male/Female (14/15)
Age (years)	60.45 ± 8.57	61.21 ± 6.40
Body height (cm)	160.84 ± 8.41	160.59 ± 8.06
Body weight (kg)	66.72 ± 9.82	70.66 ± 15.34*
BMI (kg/m^2^)	25.91 ± 3.11	27.36 ± 5.35*
Waist circumference (cm)	88.68 ± 7.93	91.32 ± 12.72*
SBP (mmHg)	130.39 ± 17.95	123.21 ± 27.86*
DBP (mmHg)	76.64 ± 10.41	72.83 ± 17.44*
PP (mmHg)	53.75 ± 15.64	50.38 ± 15.54
HbA1c (%)	6.02 ± 0.36	8.34 ± 1.78**
LDL cholesterol (mg/dL)	112.96 ± 27.47	111 ± 31.08
Blood Sugar AC (mg/dL)	109.39 ± 23.35	168.87 ± 56.76**
ECG-PWV_foot_ (m/s)	5.45 ± 0.37	5.71 ± 0.46
CT (s)	0.15 ± 0.04	0.14 ± 0.03
CTR	0.11 ± 0.02	0.11 ± 0.02
SI (m/s)	3.15 ± 0.79	3.72 ± 1.22*
f_Emax_ (Hz)	1.66 ± 0.18	2.15 ± 0.46**

Group 1 = Healthy upper middle-aged subjects, Group 2 = Diabetes mellitus subjects.

Value are expressed as mean ± SD. BMI = Body mass index; SBP = Systolic blood pressure; DBP = Diastolic blood pressure; PP = Pulse Pressure; HbA1c = Glycated hemoglobin; LDL = Low density lipoprotein; Blood Sugar AC = Fasting blood sugar; ECG-PWV_foot_ = Electrocardiography-pulse wave velocity of foot; CT = Crest time; CTR = Crest time ratio; SI = Stiffness index; f_Emax_ = Instantaneous energy of maximal energy. ^*^p < 0.05 Group 1 vs. Group 2, ^**^p < 0.001 Group 1 vs. Group 2.

### Comparison of computational parameters between diabetic and non-diabetic patients

**Figure 1:**
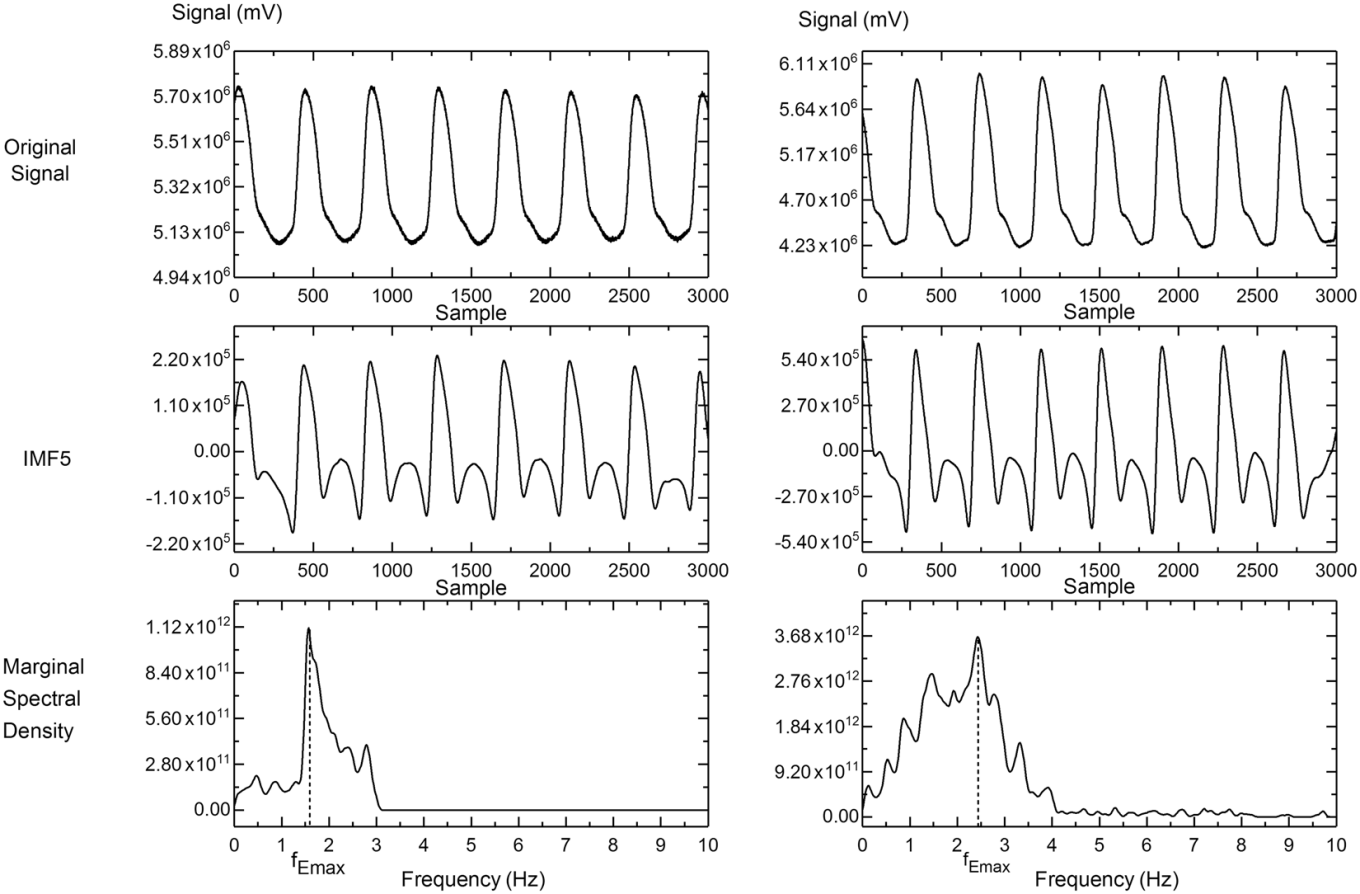
Representative illustration of original digital volume pulse (DVP) signals, intrinsic mode function 5 (IMF5) after ensemble empirical mode decomposition (EEMD), and marginal spectral density after Hilbert-Huang spectrum analysis from a healthy subject (left panel) and that from a diabetic patient (right panel), showing elevated instantaneous frequency corresponding to maximal energy (f_Emax_) in the diabetic patient compared to the healthy volunteer.

There was no significant difference in ECG-PWV_foot_, CT and CTR between subjects without diabetes (Group 1) and those with the disease (Group 2) (*p* = 0.082, 0.287, and 0.896, respectively) (Table 1). However, stiffness index (SI) and instantaneous energy of maximal energy (f_Emax_) were significantly higher in the diabetic patients (Group 2) than those in the non-diabetic subjects (Group 1) (*p* < 0.05 and <0.001, respectively) (Table 1, Fig. 1).

### Correlations between f_Emax_ and risk factors of atherosclerosis

Despite the lack of significant correlation between f_Emax_ and conventional risk factors of atherosclerosis in the non-diabetic subjects (Group 1), f_Emax_ was found to be positively associated with plasma concentration of glycated hemoglobin (*p* = 0.011) and stiffness index (SI) (*p* = 0.029) but negatively correlated with crest time (CT) (*p* = 0.015) in the diabetic patients (Group 2) (Table 2). When all testing subjects were taken into account, f_Emax_ was shown to be highly significantly associated with glycated hemoglobin and fasting blood sugar levels as well as stiffness index (all *p* < 0.001) but negatively correlated with CT (*p* = 0.017).

**Table 2:** Correlations between instantaneous frequency of maximal energy (f_Emax_) and risk factors of atherosclerosis in healthy and diabetic subjects.

	Group 1 Male/Female (14/15)	Group 2Male/Female(14/15)	Group 1 and Group 2Male/Female(28/30)
HbA1c	*r* = −0.275 *p* = 0.149	*r* = 0.472 *p* = 0.011	*r* = 0.651 *p* < 0.001
Blood Sugar AC	*r* = −0.018 *p* = 0.928	*r* = 0.337 *p* = 0.116	*r* = 0.507 *p* < 0.001
HDL cholesterol	*r* = 0.240 *p* = 0.210	*r* = 0.388 *p* = 0.074	*r* = −0.011 *p* = 0.897
LDL cholesterol	*r* = 0.357 *p* = 0.057	*r* = 0.041 *p* = 0.857	*r* = 0.081 *p* = 0.631
Triglyceride	*r* = −0.172 *p* = 0.373	*r* = −0.021 *p* = 0.927	*r* = 0.182 *p* = 0.937
TC/HDL cholesterol	*r* = −0.289 *p* = 0.128	*r* = 0.023 *p* = 0.907	*r* = 0.088 *p* = 0.827
ECG-PWV_foot_ (m/s)	r = −0.232 *p* = 0.388	r = −0.212 *p* = 0.430	r = −0.002 *p* = 0.990
CT (s)	r = −0.035 *p* = 0.858	r = −0.481 *p* = 0.015	r = −0.323 *p* = 0.017
CTR	r = 0.026 *p* = 0.895	r = −0.047 *p* = 0.822	r = −0.009 *p* = 0.948
SI	*r* = 0.234 *p* = 0.222	*r* = 0.404 *p* = 0.029	*r* = 0.440 *p* < 0.001

Group 1 = Healthy upper middle-aged subjects, Group 2 = Patients with diabetes mellitus type 2; HbA1c = Glycated hemoglobin; Blood sugar AC = Fasting blood sugar; HDL = High density lipoprotein; LDL = Low density lipoprotein; TC = Triglyceride; ECG-PWV_foot_ = Electrocardiography-pulse wave velocity of foot; CT = Crest time; CTR = Crest time ratio; SI = Stiffness index. ^*^p < 0.05 Group 1 vs. Group 2, ^**^p < 0.001 Group 1 vs. Group 2. Significance of association determined by Pearson correlation test.

## Discussion

Not only is the present study the first to apply EEMD in acquiring only six-second detrended DVP signals for assessing arterial stiffness, but it also unveiled the clinical significance of Hilbert-Huang spectrum-derived instantaneous energy of maximal energy (f_Emax_) in differentiating non-diabetic middle-aged and elderly subjects from those with diabetes by showing highly significant positive associations of f_Emax_ with key diabetes parameters and stiffness index.

Despite currently available non-invasive equipment for the assessment of vascular health, the cost of the devices and the requirement for designated personnel for operation restrict their being used as routine screening tools. For instance, the use of the air pressure sensing system (APSS) mandates the use of an inflatable pressure cuff and full assistance for measurement. By contrast, DVP acquired through photoplethysmography (PPG) is a parameter that can be acquired using simple portable devices. We have previously shown that discrepancy in DVP waveform amplitude between two upper limbs can differentiate young from aged subjects and patients with good from those with poor diabetic control as well as identify arteriosclerosis risks^15^. Using the multiscale entropy approach, DVP waveform amplitudes have also been demonstrated to differentiate healthy from diabetic subjects^16,17^.

Nevertheless, the major obstacle to the use of DVP is the noises that notably interfere with data interpretation. Ensemble empirical mode decomposition (EEMD), one of the two components of the Hilbert-Huang transformation, is an adaptive time-frequency data analysis method found to be useful for extracting signals from data produced in a variety of non-stationary and nonlinear processes^18–20^. By applying EEMD, the current study demonstrated that DVP signals can be detrended and decomposed into IMFs for arterial stiffness computation (i.e., IMF5). Moreover, the current study demonstrated that the other component of the Hilbert-Huang transformation, Hilbert-Huang spectrum, can be utilized to compute the instantaneous frequency of maximal energy (f_Emax_) that exhibited significant positive association with the parameters of acute (i.e., fasting blood sugar) and chronic (i.e., HbA1c) blood sugar control as well as the conventional stiffness indices (i.e., CT and SI).

On the other hand, the question arises regarding the cause of increase in f_Emax_ in the diabetic subjects. One plausible explanation would be the production of diabetes-associated advanced glycation end-products (AGEs) that causes collagen cross-linking in the arterial medial layer, thereby contributing to arterial stiffness^21^. The results of the present study further support the link between diabetes and arterial stiffness. Additionally, diabetes has been well-documented as a risk factor for the formation of atherosclerotic plaques that can promote turbulence in the laminar blood flow^22^. Therefore, it is rational to propose that the turbulence thus generated may partly account for the increase in f_Emax_ in the diabetic patients in the current study. The lack of significant difference in conventional parameters for assessing arterial stiffness (i.e., ECG-PWV_foot_, CT, CTR) between non-diabetic and diabetic subjects in the present study may further highlight the unique characteristic of f_Emax_ in reflecting diabetes-associated vascular dysfunction.

This study has its limitations. Firstly, the sample size of the present study is relatively small. On the other hand, the highly significant associations of f_Emax_ with the parameters of sugar control and arterial stiffness highlight the significance of our findings. Secondly, other clinical conditions associated with atherosclerosis such as hypertension, smoking, and hyperlipidemia have not been separately examined to compare their impacts on f_Emax_. Finally, there were several inherent limitations interfering with proper acquisition of DVP signal with photoplethysmography sensors. To obtain reliable data, potential interferences including skin pigmentation, tissue characteristics, blood flow in the measured area, involuntary vibrations of the subjects being examined as well as low temperature-induced peripheral vessel constriction should be minimized. Therefore, all measurements were performed in a noise-free, humidity-controlled room with the temperature maintained at 26 ± 1 °C.

In conclusion, the results of the present study showed that Hilbert-Huang transformation not only enabled the assessment of arterial stiffness from merely 6 seconds of decomposed digital volume pulse signals, but it can also produce an instantaneous frequency of maximal energy that correlated positively with the parameters of blood sugar control and conventional arterial stiffness indices. The findings shed light on the possibility of adopting this simple parameter as an index of arterial stiffness and diabetes control. The short time required for measurement and the simplicity of equipment underscore its potential widespread use in portable devices.

## Methods

### Study population

From July 2009 to October 2010, 63 middle-to-old aged men and women with and without diabetes mellitus type 2 were recruited. All diabetic patients were enrolled from the outpatient clinic for diabetes care of the Hualien Hospital. The diagnosis of diabetes was based on either a fasting glucose concentration higher than 126 mg/dL or a glycosylated hemoglobin (HbA1c) level >6.5%^23^. All patients underwent regular treatment as well as follow-up in the clinic for over two years. The criteria for enrollment of non-diabetic subjects included a negative history for diabetes mellitus, an HbA1c level less than 6.5% and a fasting glucose level lower than 126 mg/dL. Subjects with history of atherosclerosis-associated complications, such as stroke, angina, myocardial infarction and peripheral vascular diseases within three months of the present study were excluded. Of the 63 subjects recruited, five were excluded because of either inadequate length of follow-up for diabetic patients or history of known cardiovascular diseases. As a result, totally 58 subjects were recruited who were divided into two groups, including 29 non-diabetic subjects (15 females,14 males) recruited from a routine annual physical check-up program at the same hospital (Group 1) and 29 patients with diabetes mellitus type 2 (15 females,14 males) (Group 2). The protocol and procedures of this study were in accordance with the principles of the Declaration of Helsinki. The study was approved by the Institutional Review Board of Hualien Hospital. Informed consents were obtained from all study participants.

### Acquisition of anthropometric, serum biochemical, and hemodynamic parameters

All measurements and procedures were performed in the morning (i.e., 8:30–10:30 a.m.). Demographic (i.e., age, gender) and anthropometric (i.e., body weight, body height, waist circumference, body mass index) parameters, serum lipid profile (i.e., high-density lipoprotein cholesterol, low-density lipoprotein cholesterol, total cholesterol, and triglyceride), and parameters of glucose control (i.e., fasting blood glucose and glycated hemoglobin [HbA1c] levels) were obtained at the hospital. Resting blood pressure was measured once over the left arm of the supine participants using an automated oscillometric device (Microlife BP3AG1, Taiwan) with a cuff of appropriate size. Cholesterol and triglycerides concentrations were determined from blood samples obtained after overnight fasting for 12 hours. The participants were asked to refrain from caffeine-containing beverages and theophylline-containing medications for at least 12 hours before each hospital visit. Additionally, to minimize potential errors in infrared sensor readings arising from involuntary vibrations of the examinees and a decreased environmental temperature that may cause constriction of peripheral vessels, all participants received blood sampling before data acquisition and were allowed to rest in a supine position for 10 minutes in a quiet room with temperature maintained at 26 ± 1 °C. The PPG sensor was simultaneously applied to left index fingertip of each participant for the acquisition of data for 30 minutes^16,17^. From all testing subjects, 6 seconds of DVP signals were acquired after the start of recording for 5 minutes.

### Protocol of measurement of DVP and other computational parameters

The protocol for acquisition of DVP signals with photoplethysmography was described in our previous study^10^. Briefly, a self-developed six-channel electrocardiography (ECG)-PWV-based equipment was used to obtain 3000 successive DVP signals within 6 seconds from the right index finger at a frequency of 500 Hz. Infrared sensors were put on the points of reference simultaneously to acquire data. After being processed through an analog-to-digital converter USB-6009 DAQ, National Instruments, Austin, TX) with a sampling frequency of 500 Hz, the digitized signals were stored in a computer^24^. The DVP signals thus obtained were subject to two-staged Hilbert–Huang transformation (HHT) that included ensemble empirical mode decomposition (EEMD) and Hilbert–Huang spectrum.

For comparison, ECG-PWV_foot_, crest time (CT) and crest time ratio (CTR) from each subject were acquired. The computation of ECG-PWV_foot_ was conducted as previously described^14^. The time difference (ΔT) between the R peak of an electrocardiogram and the foot point of a DVP waveform during the same cardiac cycle was obtained. The distance from the sternal notch to the foot was the sum of the shortest distance from the sternal notch to medial patella, from medial patella to medial malleolus, and from medial malleolus to the tip of the second toe of each foot. ECG-PWV_foot_ was calculated by dividing the distance with ΔT and taking the average from both sides^14^. Crest time (CT) is defined as time from foot point to peak of a pulse wave, while crest time ratio (CTR) is the ratio of CT to the time from one foot point to the next foot point (i.e., cycle time)^11^.

### Hilbert–Huang transformation (HHT) of DVP signals

In general, DVP signals comprise noise-free and noise components:

1 $${\rm{y}}({t})={\rm{s}}({t})+{\rm{n}}({t})$$

in which y(*t*) is the acquired data, and s(*t*) and n(*t*) are the desired signal and white noise, respectively. As shown in (1), all data are combinations of signal and noise. To enhance the accuracy of measurements, the ensemble approach is adopted in which the data are collected by separate observations that may contain different noises. To generalize this ensemble concept, noise is introduced to the single data set y(*t*) as if different observations were actually being made as an analog to a physical experiment which could be repeated many times. Under such circumstances, the *i*th “artificial” observation will be

2 $${{\rm{y}}}_{{i}}({t})={\rm{y}}({t})+{\omega }_{{i}}({t})$$

In the case of a single observation, each multiple observation ensemble is simulated through the addition of not arbitrary but different realizations of white noise *ω*_i_(*t*) to that observation as shown in (2). EEMD decomposes the signal into different IMFs, each of which is a mono-component function^9^. Hilbert transformation is then applied to calculate the instantaneous frequencies of the original signal. After the identification of all local maxima and minima of the signal, the upper and lower envelopes are created through curve fitting. Mean values of the upper and lower envelopes of the signal, m_11_(*t*), are then obtained.

Therefore, the difference between the signal y_*i*_(*t*) and its envelopes m_11_(*t*), which is denoted as h_11_(*t*), can be computed

3 $${{\rm{h}}}_{11}({t})={{\rm{y}}}_{{i}}({t})-{{\rm{m}}}_{11}({t})$$

The approximation nature of the curve fitting method necessitates further procession of h_11_(*t*) (i.e., treating h_11_(*t*) as the signal itself with continual repetitions of the process) until satisfaction of the following two conditions^9^: (1) The number of extrema and the number of zero-crossings are either equal or differ by at most one, and (2) At all times, the mean value between the envelope defined by local maxima and that defined by local minima is zero.

Through iteration (for a total of k times), the difference between the signal and the mean envelope values, h_1*k*_(*t*), is computed as

4 $${{\rm{h}}}_{1{k}}({t})={{\rm{h}}}_{1({k}-1)}({t})-{{\rm{m}}}_{1{k}}({t})$$

where m_1*k*_(*t*) is the mean envelope value after the kth iteration, and h_1(*k*-1)_(*t*) is the difference between the signal and the mean envelope value at the (*k* − 1)th iteration. The function h_1*k*_(*t*) is then denoted as the first IMF component:

5 $${{\rm{I}}{\rm{M}}{\rm{F}}}_{1}({t})={{\rm{h}}}_{1{k}}({t})$$

After removing IMF_1_(*t*) from the original signal y(*t*), the residue can be obtained

6 $${{\rm{y}}}_{{i}}({t})={{\rm{r}}}_{1}({t})+{{\rm{I}}{\rm{M}}{\rm{F}}}_{1}({t})$$

The residue r_1_(*t*) can then be treated as the new signal, and the above iteration process is repeated to extract the rest of the IMFs from the signal y_*i*_(*t*) as:

7 $${{r}}_{{j}-1}(t)\,-\,{{\rm{I}}{\rm{M}}{\rm{F}}}_{j}(t)={{\rm{r}}}_{j}(t),j=2,\ldots ,{\rm{n}}$$

The signal decomposition process continues until r_n_(*t*) becomes a monotonic function, from which no further IMFs are extractable. The signal y_i_(*t*) is decomposed through the summation of (6) and (7) into a number of IMFs that are distinct components of the signal. Hence, the signal y(*t*) can be defined as:

8 $${\rm{y}}({t})={{\rm{I}}{\rm{M}}{\rm{F}}}_{1}({t})+{{\rm{I}}{\rm{M}}{\rm{F}}}_{2}({t})+\ldots +{\rm{I}}{\rm{M}}{\rm{F}}{\rm{n}}({t})+{{\rm{r}}}_{{\rm{n}}}({t})$$

In this way, the data are decomposed into n-empirical IMF modes and a residue, r_*n*_(*t*), which can either mean a constant or trend in (8). The concept of EEMD is to add white noise, which populates the whole time–frequency space uniformly with the components of different scales separated by a filter bank. The EEMD process is further explained as follows^9^: Step 1. Addition of a white noise series to the targeted data; Step 2. Decomposition of the data with added white noise into IMFs; Step 3. Repetition of step 1 and step 2, but with different white noise series each time; Step 4. Acquisition of the (ensemble) means of corresponding IMFs of the decompositions as the final result. When the number in the ensemble approaches infinity, the result of EEMD is obtained in which IMF_i_(*t*) + αr_*k*_(*t*) is the *k*th realization of the *i*th IMF in the signal with added noise, α is the standard deviation of the added noise, and r_*k*_(*t*) is the residual after extraction of the first k IMF components. The number of trials in the ensemble (N) needs to be large.

**Figure 2:**
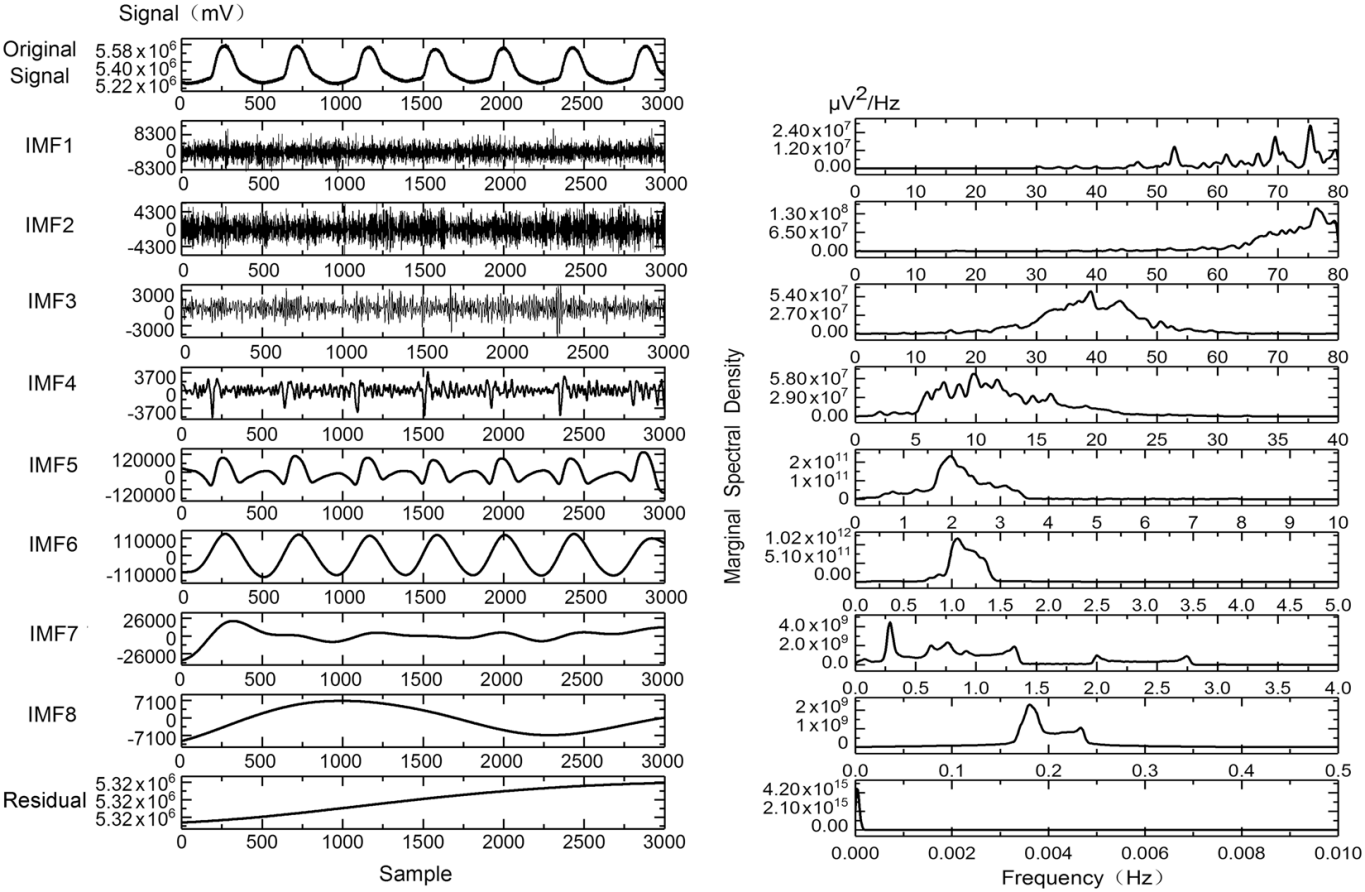
Six-second digital volume pulse (DVP) signals from a testing subject with 3000 samplings at a frequency of 500 Hz undergoing ensemble empirical mode decomposition (EEMD) resulting in the generation of 8 intrinsic mode functions (IMFs) and residual r_8_(*t*) (Left panel). Through Hilbert–Huang spectrum analysis, the instantaneous frequency corresponding to different energy can be obtained for each IMF (Right panel).

In the present study, six-second DVP signals were obtained from each testing subject with 3000 successive samplings at a rate of 500 Hz. Hence, α was set to be 0.2, and N was equal to 3000 for easy computing^20,25^. Using the Matlab package, the original 3000 samplings within the six-second DVP signals from a testing subject (i.e., y(*t*) in Eq. (1)) were subject to EEMD to obtain Eqs (2–8). The process generated 8 IMFs and residual r_8_(*t*) as illustrated in Fig. 2 (Left panel).

According to the definition of stiffness index (SI)^26^, it can be computed from IMF5(*t*) in Fig. 2 as follows:

9 $${\rm{S}}{\rm{I}}{\rm{=}}\frac{{\rm{b}}{\rm{o}}{\rm{d}}{\rm{y}}\,{\rm{h}}{\rm{e}}{\rm{i}}{\rm{g}}{\rm{h}}{\rm{t}}}{{\rm{\Delta }}{{\rm{T}}}_{{\rm{D}}{\rm{V}}{\rm{P}}}}$$

Hilbert-Huang transformation (HHT) is signal analysis in the time-frequency domain by combining EEMD with Hilbert transformation^16,19,20^. Unlike Fourier analysis that produces a series of sine and cosine functions of fixed amplitudes to represent each frequency constituent in the signal, the Hilbert–Huang spectrum approach is based on the computation of instantaneous frequency from Hilbert transformation of the signal. Generally, the definition of Hilbert transform for the signal IMFn(*t*) is

10 $${\rm{H}}{\mathtt{[}}{\rm{I}}{\rm{M}}{\rm{F}}{\rm{n}}{\mathtt{(}}{t}{\mathtt{)}}{\mathtt{]}}{\mathtt{=}}{\rm{I}}{\rm{m}}{\mathtt{(}}{t}{\mathtt{)}}{\mathtt{=}}\frac{{\mathtt{1}}}{\pi }{\int }^{}\frac{{\rm{I}}{\rm{M}}{\rm{F}}{\rm{n}}{\mathtt{(}}\tau {\mathtt{)}}}{{t}{\mathtt{-}}\tau }\,{\rm{d}}\tau $$

In theory, any complex signal z(*t*) can be considered to be the sum of its real part IMFn(*t*) and imaginary part Im(*t*) so that

11 $${\rm{z}}({t})={\rm{I}}{\rm{M}}{\rm{F}}{\rm{n}}({t})+{j}{\rm{I}}{\rm{m}}({t})$$

To be expressed in a polar coordinate system, Eq. (11) can be rewritten as

12 $${\rm{z}}(t)={\rm{\alpha }}(t){e}^{j\theta (t)}$$

where

13 $$\alpha ({t})=\sqrt{{\rm{I}}{\rm{M}}{\rm{F}}{\rm{n}}{({t})}^{2}+{\rm{I}}{\rm{m}}{({t})}^{2}}$$

14 $$\theta ({t})={\tan }^{-1}(\frac{{\rm{I}}{\rm{m}}({t})}{{\rm{I}}{\rm{M}}{\rm{F}}{\rm{n}}({t})})$$

denote the instantaneous amplitude and phase of the analytic complex signal, respectively. The instantaneous frequency ω(*t*) of the signal can be derived from the instantaneous phase θ(*t*) as

15 $${\rm{\omega }}(t)=\frac{d(\theta (t))}{dt}$$

Therefore, in terms of the amplitude and instantaneous frequency, the real part of the signal IMFn(*t*) can be written as a time-dependent function

16 $${\rm{IMFn}}(t)=\mathrm{Re}\{z(t)\}=\mathrm{Re}{\sum }_{i=1}^{n}a(t){e}^{j\theta (t)}$$

where Re{z(*t*)} represents the real part of the complex signal z(*t*). To ensure that the instantaneous frequency obtained from Eq. (15) is physically meaningful, the instantaneous phase θ(*t*) must be a mono-component function (i.e., a single-valued function over time).

As shown in (13) and (14), the instantaneous phase θ(*t*) is derived from IMFn(*t*) and its Hilbert transform. Therefore, IMFn(*t*) must also be a monocomponent function. Eq. (13) allows the amplitude and the instantaneous frequency to be presented in a 3-D plot, in which the amplitude is represented by the height in the time–frequency plane. This time–frequency distribution thus generated is known as “Hilbert–Huang spectrum” H(*ω*, *t*):

17 $${\rm{H}}(\omega {,}{t})={\rm{R}}{\rm{e}}{\sum }_{{i}=1}^{{n}}{a}({t}){{e}}^{{j}\int \omega ({t}){d}{t}}$$

On the other hand, the marginal spectrum, h(*ω*), can be defined as (Fig. 2, right panel).

18 $${\rm{h}}(\omega )={\int }_{0}^{{\rm{T}}}{\rm{H}}(\omega ,{t}{\mathtt{)}}{d}{t},$$

### Statistical analysis

Statistical analyses were performed with SPSS 14.0 (SPSS Inc., Chicago, IL, USA) including coefficient of variation, t test, and correlation. Data were expressed as mean ± standard deviation (SD). The continuous variables between the two groups were compared using two-tailed t-test. Correlations between instantaneous frequency (f_Emax_) and risk factors was analyzed with Pearson correlation test. A p value < 0.05 was considered statistically significant.

## Data Availability

The datasets generated and analyzed during the current study are available from the corresponding author on reasonable request.
